# Primary Sinonasal Lymphoma: A Rare Cause of Cranial Neuropathies

**DOI:** 10.7759/cureus.8135

**Published:** 2020-05-15

**Authors:** Raman J Sohal, Sylva Bem, Diana M Gilligan

**Affiliations:** 1 Internal Medicine, State University of New York (SUNY) Upstate Medical University, Syracuse, USA; 2 Pathology, State University of New York (SUNY) Upstate Medical University, Syracuse, USA; 3 Medicine/Hematology, State University of New York (SUNY) Upstate Medical University, Syracuse, USA

**Keywords:** non-hodgkin lymphoma, primary sinonasal lymphoma, cranial neuropathy, diplopia, dysphagia, proptosis, r-chop

## Abstract

Primary sinonasal lymphomas are a rare type of non-Hodgkin lymphoma (NHL) with an overall incidence of about 1% of all head and neck cancers. Diffuse large B-cell lymphoma (DLBCL) is the most common type of NHL and it most commonly occurs in elderly men. The diagnosis of such a lymphoma is difficult because of its varied presentation, which usually occurs late after a significant mass effect has taken place. Symptoms vary significantly, from simply nasal obstruction or epistaxis to varied cranial neuropathies or the confusion seen with central nervous system (CNS) spread. Patients may present with a large orbital mass and proptosis. Therefore, emphasis should be placed on earlier detection by using appropriate imaging modalities to reveal such masses. A biopsy is necessary to confirm the diagnosis. The prognosis is scored by the International Prognostic Index. Staging scans with whole-body computed tomography (CT) with contrast and positron emission tomography-fluorodeoxyglucose (PET-FDG) are required to determine other areas of involvement. Treatment is with R-CHOP (rituximab, cyclophosphamide, doxorubicin, vincristine, and prednisone) with the case-dependent use of intrathecal chemoprophylaxis (methotrexate) to prevent CNS spread. Here, we present a case series of two patients who were found to have a primary sinonasal lymphoma of the DLBCL type. In both cases, the presenting symptoms were vague. A high index of suspicion is required to diagnose NHL early on, which portends the best chance of a successful outcome. This article seeks to emphasize the role of including primary sinonasal lymphoma as a differential in the presentation of unrelenting cranial neuropathies or facial mass.

## Introduction

Primary sinonasal lymphomas are rare. Non-Hodgkin lymphoma (NHL) makes up 1% of all head and neck cancers. Extranodal NHL is not uncommon with an occurrence of about 40%, usually involving the gastrointestinal tract, bone, soft tissue, and dura. However, the sinuses and nasal cavity are rarely involved as a site of primary NHL lymphoma [1-2]. A review of the literature shows that the B-cell type are the most common type of NHL of the sinonasal tract [3]. The reported incidence is between 1% and 5%. Symptoms are often vague, which delays diagnosis. Symptoms are related to the site of occurrence, the size of the lesion, and the degree of expansion and invasion into local tissue. Typical symptoms often include nasal obstruction or epistaxis [4]. However, patients can also present with multiple different cranial neuropathies, including diplopia, dysphagia, and local mass effect symptoms, including proptosis. Of the sinuses, the most common location is the maxillary followed by the ethmoid, sphenoid, and frontal sinus [5]. Since the presentation of such a mass is uncommon, diagnosis is often delayed until more prominent features develop from the mass effect such as proptosis, eye swelling, and blurred vision. Often, if there is unilateral facial paralysis, it is overlooked as Bell’s palsy secondary to Lyme disease. We present two cases of primary sinonasal lymphoma. In both cases, symptoms were related to mass effect compression.

## Case presentation

Case one

We present a case of a 76-year-old male with a past medical history most significant for myasthenia gravis, rheumatoid arthritis, coronary artery disease (CAD) s/p percutaneous coronary intervention (PCI), type 2 diabetes, and atrial fibrillation on anticoagulation, who presented to the emergency department (ED) for severe left hip pain and blurred vision. His recent surgical history included a left total hip arthroplasty (THA) infection status post explantation and replantation five months later complicated by yet another repeat infection, this time undergoing Girdlestone resection arthroplasty. The patient has been non-ambulatory since.

**Figure 1:**
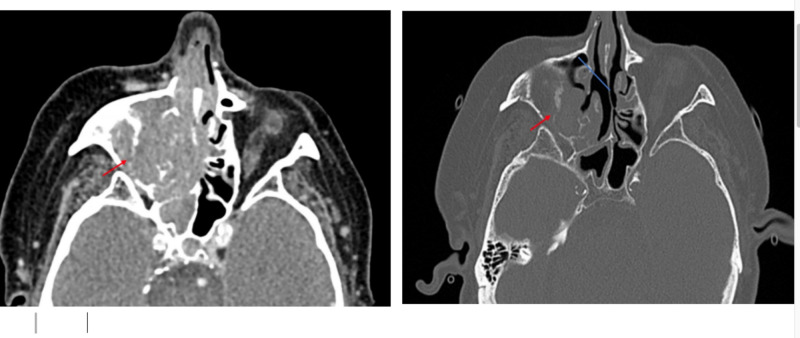
Case one computed tomography Left: Before treatment; red arrow denotes mass; Right: Two months after starting treatment

**Figure 2:**
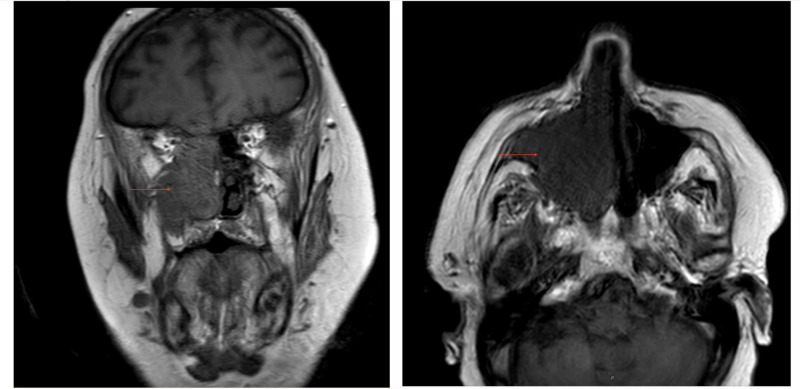
Case one magnetic resonance imaging face with and without contrast Left: coronal plane; red arrow points to mass; Right: axial plane; red arrow points to mass

On admission, the patient reported double vision and bilateral retro-orbital headache, jaw pain, and decreased appetite. He denied flashes, floaters, scalp tenderness, anosmia, nasal obstruction, fever, chills, or weight loss. Physical exam was remarkable for partial right ophthalmoplegia; restricted downward gaze, exophthalmos, bilateral infraorbital ecchymosis, and horizontal diplopia, as well as a palpable and tender right inferomedial orbital mass. There was significant edema along the right nasal wall. The left external nare appeared benign. No obvious lesion was seen on a brief view, external rhinoscopy. Bilateral auricles were unremarkable. Given these findings, there was a concern for orbital cellulitis. The patient was started on broad-spectrum antibiotics, and given his history of an immunocompromised state with type 2 diabetes and rheumatoid arthritis on methotrexate and prednisone, the patient was started on antifungal treatment with bacterial, fungal, and viral cultures sent out. The patient had seen an ophthalmologist in the outpatient setting, at which time he had also reported diplopia. The exam for visual acuity showed 20/80 OD 20/20 OS, which was corrected to 20/30, 20/20. ARMD OD > OS. There was no proptosis or exophthalmos at that time. The fundus exam was unchanged from the prior evaluation. Computed tomography (CT) head and CT maxillofacial head showed a large destructive sinus tract lesion throughout the right ethmoid, frontal, maxillary, and sphenoid sinuses with the destruction of the lamina papyracea and floor of the right orbit extending into the right inferomedial portion of the orbit (Figure 1A). Mass effect was noted along the posteromedial margin of the right globe causing mild proptosis and concern for optic nerve tension. There was also suspicion for vasogenic edema within the right frontal lobe and erosion/destruction of the right side of the cribriform plate. This was followed up with MRI, which showed focal enhancement along the floor of the anterior cranial fossa with possible tumor extension into the inferior right frontal lobe. See Figure 2. Of note, imaging from four years prior, including CT head, and magnetic resonance imaging (MRI) brain showed maxillary mucous retention cysts, right greater than left, but no other sinus disease.

**Figure 3:**
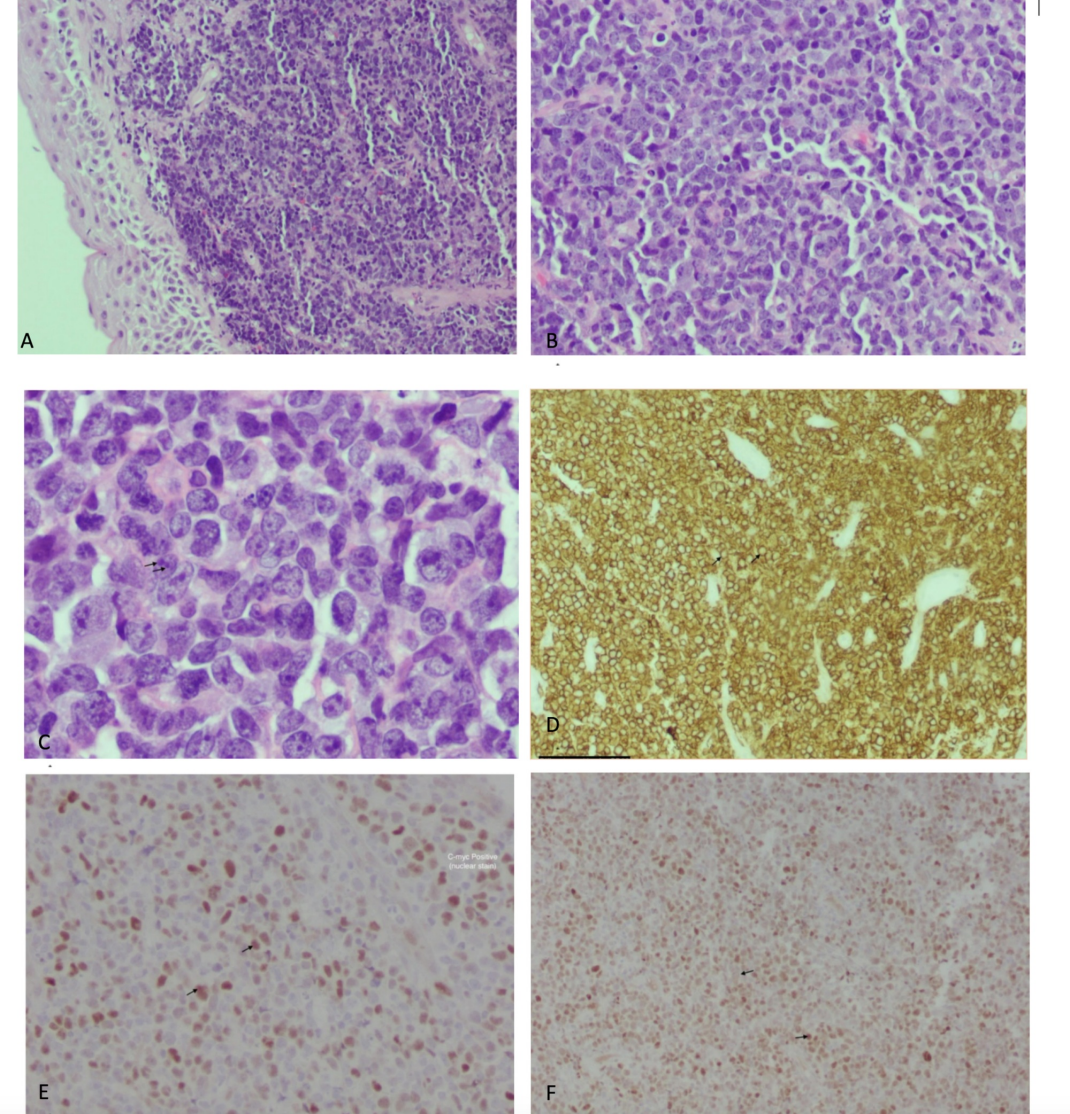
Case one stained biopsy sections A: Squamous cell epithelium with underlying malignant cell infiltration; B: High-power image of A; note degree of atypia, 0-4 nucleoli, and a moderate amount of cytoplasm; C: Note the multiple irregular nucleoli; D: Diffuse positive for cluster of differentiation 20 (CD20); E: C-myc positive (nuclear stain); F: Diffusely Bcl-6 positive

The ears, nose, and throat (ENT) team performed a biopsy that was positive for high-grade B-cell lymphoma. Tumor cell markers were positive for cluster of differentiation 20 (CD20), c-myc, and bcl-6; negative for pancytokeratin AE1/AE3, P63, CK5/6, P16, chromogranin, and synaptophysin. Figure 3 shows hematoxylin and eosin-stained sections of the tumor at low (A), medium (B), and high (C) power. Panels D, E, and F demonstrate positive staining for CD20, c-myc, and bcl-6.

Staging scans, including CT thorax, abdomen, and pelvis, showed no evidence of disease spread. Bone marrow biopsy was negative for marrow involvement. The patient completed 600 cGy RT in three fractions on 10/30/19. For the aggressive lymphoma, the patient was started on R-mini CHOP (Rituximab and reduced dose cyclophosphamide, doxorubicin, oncovin (vincristine), and prednisone (CHOP)).

Case two

We next present a case of a 70-year-old female with a past medical history of hypothyroidism, most significant for breast cancer s/p lumpectomy, who presented to the ED for dysphagia to solids as a transfer from an outside hospital for further workup. She was diagnosed with trigeminal neuralgia from Bell’s palsy about eight months ago and was treated with antibiotics and steroids after which she improved. She then had a return of symptoms three weeks later. At this time, she also developed horizontal diplopia, which was constant throughout the day. She reported frontal headache and a 35-pound weight loss over the past eight months. She denied fever, chills, or arthralgia. Videofluoroscopy showed severe pharyngeal phase dysphagia, absence of anterior hyoid movement, reduced tongue retraction, reduced laryngeal elevation, and absent epiglottic inversion.

She was admitted to the neurology service due to the progressive involvement of multiple cranial nerves (CN), including bilateral VI (with right greater than left) and left V, VII, VIII, IX, X. The physical exam showed normal optic head appearance bilaterally with no afferent pupillary defect. Limited bilateral ocular abduction, with intact adduction and vertical eye movements, were noted. Hyperesthesia was noted in the left CN V1 and V2 dermatome. Left lower motor neuron CN VIII was affected, with reduced hearing to finger rub. Tongue deviation to the right was seen. There was a weakened gag and swallow reflex with hoarseness of voice. CN XII exam of the sternocleidomastoid and trapezius muscle was intact bilaterally. Upper and lower extremity strength and tone were symmetric bilaterally. There was no ataxia or dysmetria. Reflexes reduced bilaterally in the upper extremity. Lumbar puncture was done. Cerebrospinal fluid (CSF) analysis showed elevated protein with normal opening pressure. The study was negative for neuromyelitis optical (NMO) antibody, CSF fungal culture, CSF autoimmune encephalitis panel, and serum anti Gq1b panel. The serum autoimmune panel was also negative. Erythrocyte sedimentation rate (ESR) was elevated at 70 mm/hr and C-reactive protein (CRP) was elevated at 28 ml/L. Laboratory exam for herpes simplex virus (HSV)-1/2, Cryptococcus, cytomegalovirus (CMV), varicella-zoster virus (VZV), streptococcal (agalactiae, pneumoniae), Neisseria meningitides, Listeria monocytogenes, and fungal cultures were negative. Cytology was negative for malignancy. Flow cytometry was negative for a lymphoproliferative disorder.

**Figure 4:**
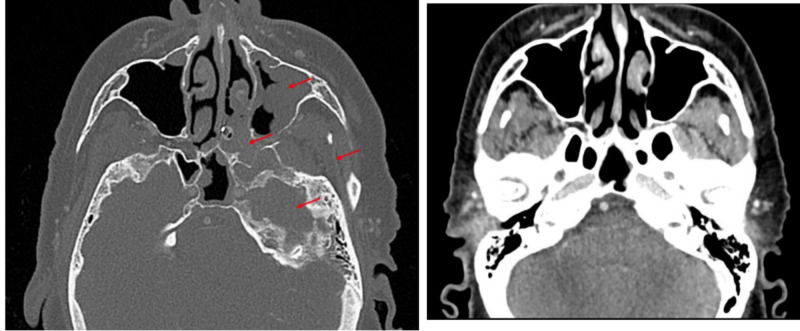
Case two computed tomography Left: Red arrows denote mass; Right: After two months of treatment

CT head showed a mass involving the left masticator space and pterygopalatine foramen with the destruction of the left pterygoid and extension into the posterior superior aspect of the left maxillary sinus as well as the posterior left nasal choana (Figure 4). The mass also extended intracranially through the left foramen rotundum to involve the left cavernous sinus, Meckel's cave, and left sphenoid cell. The mass involved the skull base left more than right with the elevation of the clivus and the involvement of the left jugular foramen. The cephalocaudal extent of the mass, including the cavernous sinus and extracranial component, measured approximately 4.6 cm and the anteroposterior dimension at least 4.3 cm. There was also an epidural extension of the mass along the dorsal aspect of the clivus. Follow-up MRI with the skull base protocol showed extensive skull base abnormality arising from the moderately enhancing mass in the left pterygopalatine fossa. The mass extended into the posterior left nasal cavity and focally into the posterior left maxillary sinus. The mass involved a large part of the left masticator space with the involvement of the left V2/foramen rotundum and left V3/ foramen oval, as well as the left facial nerve. Apparently, through perineural spread, there was an intracranial extension with the involvement of the left cavernous sinus, bilateral Meckel's caves, as well as extensive involvement of the posterior skull base, including the left jugular foramen and clivus.

**Figure 5:**
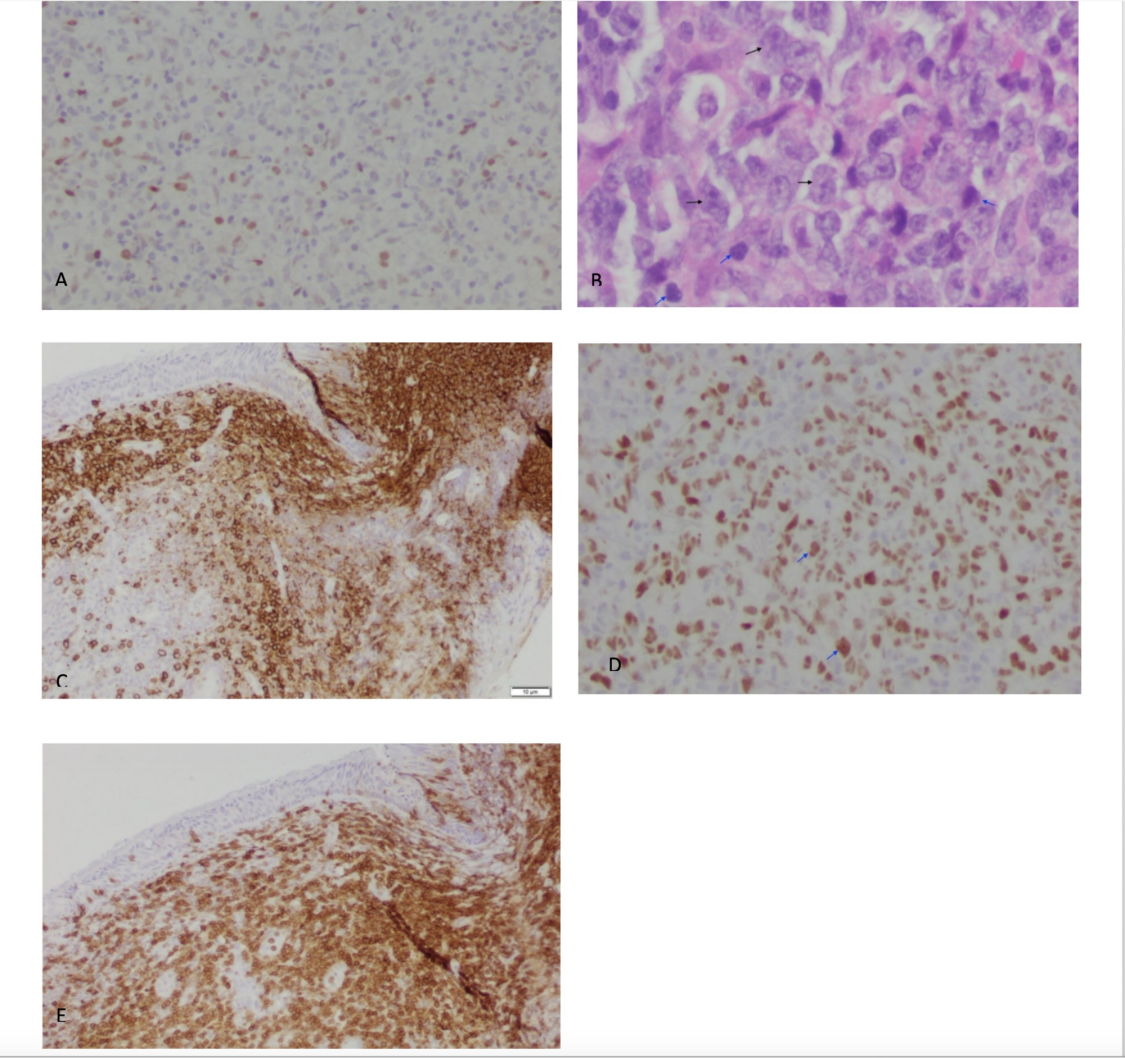
Case two stained pathology specimens A: C-myc negative. High power; B: Malignant cells with high nuclear to cytoplasmic ratio; C: CD20 positive; D: Immunostain Bcl-6 positive; E: CD3 + T-cell infiltration CD: cluster of differentiation

The nasopharyngeal biopsy was positive for diffuse large B-cell lymphoma - germinal center type (Figure 5). The biopsy showed focal nodular areas with mostly large cells suggestive of follicular lymphoma, grade 3B with partial transformation into diffuse large B-cell lymphoma. Fluorescence in situ hybridization (FISH) for t(14;18) was negative. The abnormal cells were CD20 positive Bco-expressed CD10 (weak), Bcl-6, and C-MYC (partial) but were negative for Mum-1, Bcl-2, CD30, ALK, CD21, and CD25. Ki-67, a marker of proliferation, was high (Figure 5). Stained sections were from a biopsy of case two The patient was started on R-CHOP therapy for a total of five months. She underwent six cycles of R-CHOP (rituximab 375 mg/m^2^ IV, cyclophosphamide 750 mg/m^2^, vincristine 2 mg IV, doxorubicin 50 mg/m^2^) along with intrathecal methotrexate (total of three doses each at 12 mg). She also underwent radiation to the base of the skull for a total of 25 days. Repeat imaging scans, including CT thorax, CT abdomen and pelvis, and CT soft tissue neck without contrast were performed every six months. No evidence of disease progression or abnormal enhancement of the brain was seen. The clivus showed posttreatment changes but no changes in the masticator or parapharyngeal spaces.

## Discussion

Diffuse large B-cell lymphoma is the most common type of non-Hodgkin lymphoma in the western world involving the head and neck. There is a male predominance and the most common age group is the elderly with age >60. The International Prognostic Index (IPI) is the most commonly used staging system for DLBCL [4-6]. The standard of care is chemotherapy with R-CHOP. PNL has a high risk of CNS involvement and intrathecal methotrexate (MTX) can be used to prevent CNS spread [7-8]. These cases demonstrate a rare presentation of a primary NHL of the head and neck. The symptoms usually present as a consequence of an enlarging mass leading to a local compressive effect on the surrounding tissue. In the first case, the major presenting symptom was of blurred vision and diplopia along with an infraorbital mass. The second case presented as dysphagia and multiple cranial neuropathies. Differential diagnoses included a parenchymal brainstem lesion such as glioma, lymphoma, Chronic Lymphocytic Inflammation with Pontine Perivascular Enhancement Responsive to Steroids (CLIPPERS), demyelinating, rhomboencephalitis, or neuro-Bechet. A meningitic process, such as carcinomatosis, atypical meningitis, neurosarcoidosis, and idiopathic hypertrophic cranial pachymeningitis, was considered as well. A disease process producing multiple cranial neuropathies, such as human immunodeficiency virus (HIV), Guillian Barre syndrome variant, or a skull-based tumor, was also considered. Common to both of these cases was the role of imaging in elucidating a mass involving the sinuses and skull base. These lesions were then biopsied, which revealed the diagnosis of DLBCL. In both cases, the patients were elderly and had underlying immunologic disease or cancer history. The patient in case one had rheumatoid arthritis and myasthenia gravis and had been on immunosuppression with MTX and prednisone. The patient in case two had a history of breast cancer that was treated with chemotherapy and lumpectomy. The IPI for DLBCL score was 4 points for both patients, which meant they were in the high-risk group (59% overall survival), with about a 50% chance of progression-free survival. In both patients, further scanning images revealed no additional sites of extranodal NHL involvement, which conferred a better prognosis. Intrathecal chemoprophylaxis with MTX was given to prevent leptomeningeal spread in both patients.

Although rare, NHL should be included in the differential diagnosis of soft tissue tumors of the sinonasal tract, especially in patients in their 60s-70s [9]. Obstructive symptoms are not always present and the prognosis is variable, depending on the stage and the aggressiveness of the tumor. It is important to keep a broad differential in the workup of a sinus tract mass with a biopsy to determine the source [10-11]

## Conclusions

To conclude, NHL should be included in the differential diagnosis of soft tissue tumors of the sinonasal tract, especially in the age group of 60 to 70. While obstructive symptoms are not always present and the prognosis is variable, it is important to keep a broad differential in the workup of a sinus tract mass with a biopsy. The role of CSF prophylaxis is variable with regard to the treatment of DLBCL, but it was used in both our patients, and they remain free of CNS spread.
